# A Chemo-Mechanical Model of Diffusion in Reactive Systems

**DOI:** 10.3390/e20020140

**Published:** 2018-02-22

**Authors:** Kerstin Weinberg, Marek Werner, Denis Anders

**Affiliations:** 1 Chair of Solid Mechanics, Faculty IV, Department of Mechanical Engineering, University of Siegen, Paul-Bonatz-Str. 9-11, 57076 Siegen, Germany; 2 Computational Mechanics and Fluid Dynamics, Faculty of Computer Science and Engineering, TH Köln/ University of Applied Science, Claudiusstrasse 1, 58076 Köln, Germany

**Keywords:** reaction-diffusion systems, chemical reaction, elasticity, multi-component system, finite deformations, phase decomposition, phase field model

## Abstract

The functional properties of multi-component materials are often determined by a rearrangement of their different phases and by chemical reactions of their components. In this contribution, a material model is presented which enables computational simulations and structural optimization of solid multi-component systems. Typical Systems of this kind are anodes in batteries, reactive polymer blends and propellants. The physical processes which are assumed to contribute to the microstructural evolution are: (i) particle exchange and mechanical deformation; (ii) spinodal decomposition and phase coarsening; (iii) chemical reactions between the components; and (iv) energetic forces associated with the elastic field of the solid. To illustrate the capability of the deduced coupled field model, three-dimensional Non-Uniform Rational Basis Spline (NURBS) based finite element simulations of such multi-component structures are presented.

## 1. Introduction

Specifically designed multi-component materials become more and more important in many technical applications. The functional material properties are often provided by means of chemical reactions. In fact, there is a huge range of desired and undesired chemical reactions in multi-component systems, e.g., the corrosion of metal alloys, the combustion of propellants, the discharging process in batteries or photochemical reactions in bio-chemistry, to name a few. Because the main feature of chemical reactions is their ability to generate substances which have different properties than their base products, some of these reactions can be exploited for micro-structural optimization of the multi-component system.

The microstructural arrangement of the material determines the structural properties, the life expectation and the reliability of the whole system. Therefore, there is a need for a profound knowledge of the processes ongoing in multi-component reaction-diffusion systems. Many scientific approaches to diffusive and reactive media are based on the pioneering works of Kolmogorov [1] and Fisher [2] on non-linear diffusion equations and their solutions. The analysis of structure, formation and dynamics of patterns in reactive media leads to specific rate equations and stability criteria [3,4]. The acquired mathematical knowledge on such systems is too extensive to be discussed here, we refer to [5] and references therein.

The aim of our work is to model solid mechanical structures which undergo elastic and inelastic deformations coupled with diffusion processes and chemical reactions. Here, the knowledge on reaction-diffusion systems is rather rare and always restricted to small deformations and linearized elasticity. A first formulation of diffusion equations expressing mass continuity under chemical reactions can be found in the book of de Groot and Mazur [6], generalized diffusion models that incorporate elasticity are typically based on Larchté and Cahn’s work [7]. To the best of the author’s knowledge there is no work on reaction-diffusion systems undergoing large deformations.

For this reason, we propose here a chemo-mechanical model of diffusion in reactive multi-component systems. For the derivation we stay within the framework of non-equilibrium thermodynamics, cf. [6]. A useful tool to get insight into the complicated mechanisms of such coupled systems are computational simulations. Therefore we focus on a thermodynamically sound and efficient model which captures the essential features of the multi-field system but does not necessarily include all aspects connected with specific chemical reactions.

The rest of the paper is organized as follows. In the next Section 2, we state the basic relations of finite deformation elasticity and diffusive flux. In Section 3, the fundamentals of chemical reactions are added and the coupled elastic multi-component reaction-diffusion system is formulated. Section 4 is devoted to sample computations performed with a Non-Uniform Rational Basis Spline (NURBS) based finite element analysis. A short summary in Section 5 concludes the paper.

## 2. Mechanical Deformation and Diffusion

In this section we outline the coupled chemo-mechanical model of diffusion in reactive systems. We begin with the kinematics of finite deformations and then provide the basic equations that govern the elastic field, the diffusive flux and the chemical reactions.

### 2.1. Kinematics of Deformation

For definiteness, we assume the system to be solid-state. A point in its material configuration $\Omega_{0}\subset I\;R^{3}$ is located at $X={\left(X_{1},X_{2},X_{3}\right)}^{T}$ and after a period of time $t\in I\;R^{+}$ the material point is located at position x(X,t) of the current configuration $\Omega_{t}\subset I\;R^{3}$. The corresponding deformation mapping $\Phi \left(X,t\right):\Omega_{0}\times \left[0,T\right]\to \Omega_{t}$ is uniquely described by its material gradient,

(1) $$F\left(X,t\right)=\nabla \Phi \left(X,t\right)\;.$$

For the chemo-mechanical system we assume a state in which the action of the elastic field, the transport of mass, the rearrangements of phases and chemical reactions may induce elastic and inelastic deformations. Consequently, the deformation gradient (1) is composed of elastic and inelastic parts, $F=F^{e}F^{i}$. This multiplicative decomposition is a convenient mathematical representation for a system undergoing multiphysics processes in large deformations, cf. [8]. The local inelastic component $F^{i}$ may be a result of the intercalation of particles and of chemical reactions, which generate substances with volume different from their reactants, whereas the elastic component $F^{e}$ follows from the underlying assumption of energy optimization in the system.

In the following we assume the geometrical changes of the initial configuration induced by particle diffusion, intercalation and chemical reactions to be purely volumetric and to solely depend on the local concentration c∈[0,1] in the multi-component system. This assumption excludes effects of local thermal expansion as well as microscopic realignments as induced, e.g., by first order phase transitions. We denote the corresponding volumetric component as $J^{i}=\det F^{i}$. Then, the multiplicative decomposition of the deformation gradient (1) into elastic and inelastic components simplifies to

(2) $$F={J^{i}}^{\frac{1}{3}}F^{e}\;,$$

and for the volumetric component of the deformation it follows $J=\det F=J^{e}J^{i}$.

The induced volume change can be approximated by the partial molar volume $V^{m}$ of the substances weighted with their concentration $c_{k}$. Here, we normalize the partial molar volume of the *k*th specimen with the total one, $V_{k}=V_{k}^{m}/\left(\frac{1}{n}\sum_{k=1}^{n}V_{k}^{m}\right)$ to obtain the volume change induced by the rearrangements of the diffusing specimens. According to this definition, the volumetric deformation component can be calculated by

(3) $$J^{i}=\sum_{k=1}^{n}V_{k}c_{k}.$$

The isochoric part of the deformation equals that of the elastic deformation, $\bar{F}={\bar{F}}^{e}$, and so does the right Cauchy-Green tensor, $C=F^{T}F$ and its isochoric part,

(4) $$\bar{C}={\bar{C}}^{e}=J^{-2/3}F^{T}F\;.$$

### 2.2. Mass Balance

We consider a general multi-component system consisting of *n* components. Neglecting chemical reactions at the moment, i.e., if the mass is conserved, the temporal evolution of the concentration fields $c_{k}$ is driven by a diffusive mass current $J_{k}$ according to

(5) $$\rho \frac{dc_{k}}{dt}=-\nabla \cdot J_{k}\;\;k=1,\ldots ,n$$

with

(6) $$\rho =\sum_{k=1}^{n}\rho_{k}\;,$$

where $\rho_{k}$ is the mass density of the *k*th component and the mass concentration with respect to the material configuration is defined as

(7) $$c_{k}=\frac{\rho_{k}}{\rho }.$$

Please note that $d\left(•\right)/dt=\partial \left(•\right)/\partial t+v\cdot \nabla \left(•\right)$ denotes the substantial time derivative with the barycentric velocity field v. The scalar fields $\rho_{k}$ and $c_{k}$ characterize the mass and mass fractions of the respective material components. For the transformation from the material into the spatial configuration and vice versa the usual rules apply, e.g., two fictious co- and contravariant spatial vectors a and b relate to their material counterparts by $A=F^{-T}a$ and $B=JF^{-1}b$, respectively.

In the case of the generalized Fickian diffusion Equation (5), the diffusive mass current $J_{k}$ of component *k* follows from the gradient of a chemical potential

(8) $$J_{k}=-\rho M\nabla \mu_{k}\;,$$

where M is a mobility tensor which, in general, differs for every component, phase and direction. The chemical potential, in turn, is derived from the system’s Helmholtz free energy.

### 2.3. Helmholtz Free-Energy Density of the System

The Helmholtz free energy involves elastic, configurational and interfacial energy contribution

(9) $$F_{0}\left(F,c_{1},c_{2},\ldots ,c_{n},\nabla c_{1},\nabla c_{2},\ldots ,\nabla c_{n},T\right)=RTy_{0}\int_{\Omega_{0}}\left(\Psi^{\mathrm{ela}}+\Psi^{\mathrm{con}}+\Psi^{\mathrm{int}}\right)\;d\Omega$$

and refers to the material configuration of the system. For simplicity we assume ambient conditions with constant pressure which allows to formulate the free energy (9) in equivalence to the Gibbs free energy. Expression (9) is normalized by the product of universal gas constant R, temperature *T*, and a reference molar concentration $y_{0}$, to render the overall energy density $\Psi =\Psi^{\mathrm{ela}}+\Psi^{\mathrm{con}}+\Psi^{\mathrm{int}}$ dimensionless.

#### 2.3.1. Elastic Energy Contribution

For the chemo-mechanical model we consider an elastic energy per unit undeformed volume,

(10) $$\Psi^{\mathrm{ela}}=J^{i}\left(\frac{1}{4}K^{*}\left(J^{e^{2}}-1-2\ln J^{e}\right)+\frac{1}{2}G^{*}\left(\mathrm{tr}{\bar{C}}^{e}-3\right)\right)\;.$$

Here, $K^{*}=K^{*}\left(c_{1},c_{2},\ldots ,c_{n},T\right)$ and $G^{*}=G^{*}\left(c_{1},c_{2},\ldots ,c_{n},T\right)$ denote normalized functions of the elastic bulk modulus *K* and the shear modulus *G*, respectively. Both elastic moduli depend on the concentration of the components and also on the temperature.

The elastic energy density (10) accounts for the elastic field as a consequence of particle diffusion. Exemplary we recall here the process of charging and discharging of silicon anodes in lithium batteries, where a volume expansion of more than 300% is observed due to the lithium ion intercalation [9,10]. Even if this case is extreme, a general approach to volume changes requires a finite-deformation kinematic and a hyperelastic strain energy density as stated in (10).

#### 2.3.2. Configurational Energy Contribution

The configurational free energy density $\Psi^{\mathrm{con}}$ for a multiphase mixture is given by

(11) $$\Psi^{\mathrm{con}}=\sum_{i=1}^{n}g_{i}^{0}\left(T\right)c_{i}+\theta \left(T\right)\sum_{i=1}^{n}c_{i}\ln c_{i}+\frac{1}{2}\sum_{\begin{array}{c} 1\leq i,j\leq n\\ i\neq j \end{array}}c_{i}c_{j}\chi_{ij}^{\left(0\right)}\;,$$

where $g_{i}^{0}\left(T\right)$ are normalized Gibbs free energy densities of the pure *i*th component and the second term accounts for energy contributions from the entropy of mixing. The entropy contribution is typically weighted by the temperature dependent material parameter θ. The first two energy contributions represent the classical Lewis-Randall ideal solution model. For the consideration of realistic solutions, which generally show a non-ideal behavior, it is essential to include a third term, a Porter type excess energy contribution, into the configurational energy representation [11]. The excess energy describes the decomposition of a non-ideal mixture into distinct phases with material-specific and symmetric interaction parameters $\chi_{ij}$.

Exemplary we consider a binary mixture with two equilibrium phases at the concentration $c_{\alpha }$ (α-phase) and $c_{\beta }$ (β-phase). With $c\equiv c_{2}=1-c_{1}$, θ=1, and a similar Gibbs free energy of the components, the configurational free energy density simplifies to

(12) $$\Psi^{\mathrm{con}}=c\ln\left(c\right)+\left(1-c\right)\ln\left(1-c\right)+\chi c\left(1-c\right)\;.$$

**Figure 1:**
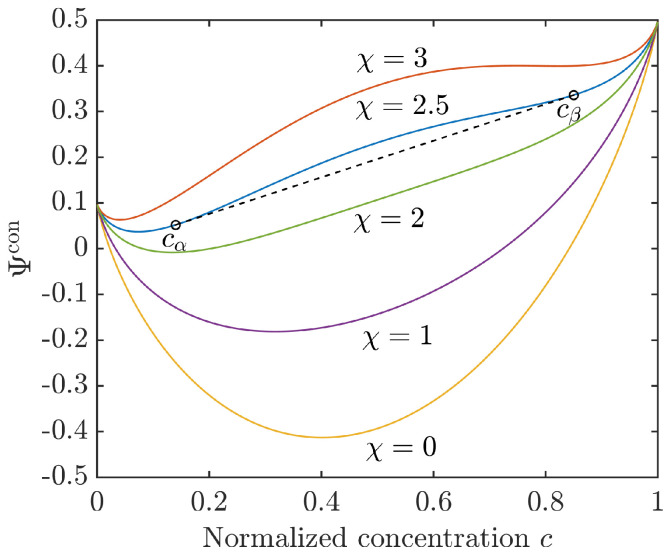
Configurational energy density (12) for different Flory–Huggins interaction parameters χ with $g_{1}^{0}=0.1$ and $g_{2}^{0}=0.5$. For χ=2.5 the common tangent indicates the equilibrium concentrations $c_{\alpha }$ and $c_{\beta }$.

For a Flory–Huggins interaction parameter χ<2 the mixing energy (12) is convex and corresponds to the energy of a homogeneous solution. Phase separation will be observed only for χ>2, when the configurational energy turns into a double-well function, i.e., it has two relative minima and a concave (spinodal) region in between. The concentrations $c_{\alpha }$ and $c_{\beta }$, i.e., the binodal points, can be determined by the common tangent rule, $\partial_{c}\Psi^{\mathrm{con}}\left(c_{\alpha }\right)=\partial_{c}\Psi^{\mathrm{con}}\left(c_{\beta }\right)$. The situation is illustrated for different values of χ in Figure 1.

#### 2.3.3. Interfacial Energy Contribution

The co-existence of phases causes additional energy contributions. This interfacial free energy density $\Psi^{\mathrm{int}}$ accounts for the nonlocal effect of surface tension which is related to surface energy density contained within the interfacial regions between different phases. It is formally given by

(13) $$\Psi^{\mathrm{int}}=\sum_{i=1}^{n}\frac{\kappa_{i}}{2}{∥\nabla c_{i}∥}^{2}\;,$$

where the material parameters $\kappa_{i}$ are related to surface energy density and thickness of the interfacial layers between the phases.

### 2.4. Mechanical Stresses and Chemical Potential

With contributions (10), (11) and (13) the system’s free energy (9) reads

$$F_{0}=RTy_{0}\int_{\Omega_{0}}\Psi \left(F,T,c_{1},\ldots ,c_{n},\nabla c_{1},\ldots ,\nabla c_{n}\right)\;d\Omega$$

and the chemical potential of the *k*th component follows from its variation with respect to the corresponding concentration $\delta_{c_{k}}F_{0}$. We remark that the molar chemical potential expresses the free energy gain for adding one mole of component *k* to the system, thus being the same for any configuration. Here, we will continue to use a non-dimensional formulation, viz.,

(14) $$\mu_{k}=\delta_{c_{k}}\Psi \left(F,T,c_{1},\ldots ,c_{n},\nabla c_{1},\ldots ,\nabla c_{n}\right)\;.$$

In turn, the mechanical stresses are the work conjugate of the deformation, $\delta_{F}F_{0}$.

From Equation (9) and with the additional constraint $\sum_{i=1}^{n}c_{i}=1$, it follows

(15) $$\begin{array}{cc} \mu_{k} & =\partial_{c_{k}}\Psi^{\mathrm{con}}-\nabla \cdot \left(\partial_{\nabla c_{k}}\Psi^{\mathrm{int}}\right)+\partial_{c_{k}}\Psi^{\mathrm{ela}}\\ & =g_{k}^{0}-g_{n}^{0}+\theta \left(T\right)\ln\frac{c_{k}}{c_{n}}+\sum_{\begin{array}{c} 1\leq i\leq n-1\\ i\neq k \end{array}}c_{i}\left(\chi_{ik}^{\left(0\right)}-\chi_{in}^{\left(0\right)}\right)+\left(c_{n}-c_{k}\right)\chi_{kn}^{\left(0\right)}-\kappa_{k}▵c_{k}-\kappa_{n}\sum_{j=1}^{n-1}▵c_{j}\\ & +\frac{1}{4}\left(\left(V_{k}-V_{n}\right)K^{*}+J^{i}K{,}_{k}\right)\left({J^{e}}^{2}-1-2\ln J^{e}\right)+\frac{1}{2}\left(\left(V_{k}-V_{n}\right)G^{*}+J^{i}G{,}_{k}\right)\left(\mathrm{tr}{\bar{C}}^{e}-3\right)\;\\ & +\frac{1}{2}\left(V_{k}-V_{n}\right)K^{*}\left(1-{J^{e}}^{2}\right), \end{array}$$

where $K{,}_{k}$ abbreviates the derivative of the overall elastic bulk modulus function $K^{*}\left(c_{1},\ldots ,c_{n},T\right)$, with respect to the corresponding concentration $c_{k}$; the same holds also for $G{,}_{k}$. These terms vanish for constant elastic parameters. Obviously, the last line in (15) comprises the coupling of the mass diffusion and the elastic field.

The first Piola-Kirchhoff stress tensor follows from the free energy with contributions (10)–(13) as

(16) $$P=J^{i}\left(\frac{K^{*}}{2}\left({J^{e}}^{2}-1\right)F^{-T}+G^{*}J^{-\frac{2}{3}}\left(F-\frac{1}{3}\mathrm{tr}\left(C\right)\;F^{-T}\right)\right)\;.$$

Please note that the elastic stresses are pulled back to the material configuration. The Cauchy stress σ in the current configuration can be obtained by a push-forward $\sigma =J^{-1}FP^{T}$, cf. [12]. The coupling of diffusion and elasticity manifests itself in the concentration dependent elastic moduli. Specifically, we formulate for a binary mixture of components *A* and *B* a Vergard’s mixture rule:

(17) $$K^{*}=\frac{K_{B}}{RTy_{0}}\left(c+\frac{K_{A}}{K_{B}}\left(1-c\right)\right)\;,K{,}_{k}=\frac{K_{B}-K_{A}}{RTy_{0}}\;,$$

(18) $$G^{*}=\frac{G_{B}}{RTy_{0}}\left(c+\frac{G_{A}}{G_{B}}\left(1-c\right)\right)\;,G{,}_{k}=\frac{G_{B}-G_{A}}{RTy_{0}}\;.$$

## 3. Chemical Reactions

A chemical reaction is defined as a process that provokes an interconversion of chemical species. Such reactions may be elementary or stepwise, when reactions encompass at least one reaction intermediate and involve consecutive elementary reactions, [13] (p. 1167). The symbolic representation of a chemical reaction is typically written as an ’equation’ in which the reactant entities, multiplied by stoichiometric coefficients, are summarized on the left hand side and the product entities are written on the right hand side. Different symbols are used to connect the reactants, e.g., the symbol → specifies a net forward reaction and symbol ⇌ is used for the stoichiometric relation of an equilibrated chemical reaction.

### 3.1. Kinetics of Chemical Reactions

Exemplary we consider a typical binary chemical reaction

(19) $$n_{A}A+n_{B}B\overset{J}{\to }n_{C}C+n_{D}D\;,$$

in which $n_{A}$ moles of molecules of type *A* and $n_{B}$ moles of molecules labeled *B* react to produce $n_{C}$ moles of molecules of type *C* and $n_{D}$ moles of molecules labeled *D*. In this example, the coefficients $n_{i}$ represent the stoichiometric coefficients for molecules *A*, *B*, *C* and *D*. The reaction rate of the respective chemical reaction is symbolized by the (positive) parameter J which gives information on the kinetics of a chemical reaction. For example, corrosion (oxidation) of an alloy under ambient conditions can take many years and has a low reaction rate, whereas combustion is an extremely fast reaction with high values of J.

The reaction rate is usually modeled by a phenomenological rate law, which expresses J as a function of the amount of each reactant in a reacting mixture, giving for our model reaction (19) the rate equation:

(20) $$J=k{\left[A\right]}^{\alpha }{\left[B\right]}^{\beta }\;,$$

where $\left[A\right]$ and $\left[B\right]$ express the amount of concentration of the species *A* and *B*, respectively. The positive proportionality constant *k*, independent of $\left[A\right]$ and $\left[B\right]$, is referred to as rate coefficient. The constant exponents α and β are independent of concentration and time, cf. [13] (p. 1147). According to [14] (p. 111) the exponents in the rate equation of an elementary reaction are given by the corresponding stoichiometry factors. Thus, if we assume our reaction (19) to be elementary, it holds $\alpha =n_{A}$ and $\beta =n_{B}$. In general α and β cannot be identified with the respective stoichiometric coefficients of the balanced stoichiometric relation. Instead all parameters of the rate equation have to be determined experimentally.

Here we will focus on elementary equilibrium reactions of type

(21) $$n_{1}A_{1}+n_{2}A_{2}+\ldots +n_{m}A_{m}\overset{J^{\prime }}{⇌}n_{1}^{\prime }C_{1}+n_{2}^{\prime }C_{2}+\ldots +n_{q}^{\prime }C_{q}\;,$$

where $J^{\prime }$ denotes the overall reaction rate which follows from the difference between the forward and backward reaction rates according to

(22) $$J^{\prime }=k_{+1}\prod_{i=1}^{m}{\left[A_{i}\right]}^{n_{i}}-k_{-1}\prod_{j=1}^{q}{\left[C_{j}\right]}^{n_{j}^{\prime }}\;.$$

According to this notation $k_{+1}$ is the rate coefficient characterizing the reaction that consumes the species $A_{i}$ to produce the species $C_{j}$; whereas $k_{-1}$ characterizes the rate coefficient for the backward reaction, which consumes the quantities $C_{j}$ and in return it produces $A_{i}$ (for i=1,…,m and j=1,…,q).

### 3.2. Thermodynamical Model for Reaction-Diffusion Systems

In a reaction-diffusion system the evolution of the concentration is driven by two competitive mechanisms, namely chemical reactions and the transport processes. On the one hand, chemical reactions alter the local concentrations of their reactants and products, following the concentration dependent rate law (22). On the other hand, diffusion decreases the spatial gradients of concentration profiles in such a manner that mass transport takes place from regions of high concentration to regions of smaller concentration values.

In opposition to the mass conservation approach stated in Equation (5), we focus here on the effect of the chemical reaction. Neglecting the diffusive flux at the moment, the corresponding source term for an *n*-component system, which undergoes *r* reactions, has the form:

(23) $$\rho \frac{dc_{k}}{dt}=\sum_{j=1}^{r}v_{kj}{\bar{J}}_{j},\;k=1,\ldots ,n\;.$$

Mass density ρ and concentration $c_{k}$ are defined above, in Equations (6)–(7). ${\bar{J}}_{j}$ is a generalized reaction rate of the *j*th chemical reaction.

Please note that an application of the mass concentration as evolution variable to describe the local composition of our system, demands the classical reaction rate J, which is originally formulated in molar concentrations, to be adjusted in its dimension. To do so, a few prerequisite calculations are required.

The coefficient $v_{kj}$ divided by the molecular mass $M_{k}$ of component *k* is proportional to the stoichiometric coefficient of the *k*th chemical substance in the *j*th chemical reaction. By convention, coefficients $v_{kj}$ are counted positive if the *k*th chemical substance is produced within the *j*th chemical reaction; and negative if the *k*th substance is consumed in the reaction *j*. Since mass conservation has to be guaranteed in each separate chemical reaction, one must require

(24) $$\sum_{k=1}^{n}v_{kj}=0,\;j=1,2,\ldots ,r\;.$$

Again, we normalize the coefficients $v_{kj}$ in such a manner that for the reactants $\left(k=1,2,\ldots ,q_{j}\right)$ of each reaction *j*

(25) $$\sum_{k=1}^{q_{j}}v_{kj}=-1,\;j=1,2,\ldots ,r$$

holds. In view of the mass conservation (24) and the normalization (25) we obtain a normalization for the products $\left(k=q_{j}+1,\right.$ $\left.q_{j}+2,\ldots ,n\right)$ given by

(26) $$\sum_{k=q_{j}+1}^{n}v_{kj}=1,\;j=1,2,\ldots ,r\;.$$

According to Equation (6) we may rewrite the kinetic quantities of chemical reactions in terms of (mass) concentration. To this end we employ the relation

(27) $$\left[y_{i}\right]=\frac{\rho_{i}}{M_{i}}=\frac{\rho c_{i}}{M_{i}}\;,$$

where $\left[y_{i}\right]$ is the molar concentration of the *i*th component (in units of mol/m^3^), $\rho_{i}$ denotes the partial density of component *i* (in units of kg/m^3^) and $M_{i}$ is the respective molar mass of constituent *i* (in units of kg/mol). An application of (27) gives the true stoichiometric coefficients $n_{kj}$ and the conventional reaction rates $J_{j}$

(28) $$n_{kj}=\frac{a_{j}v_{kj}}{M_{k}}\;\;\mathrm{and}\;\;J_{j}=\frac{{\bar{J}}_{j}}{a_{j}}\;,$$

where the proportionality constants $a_{j}$ (in units of kg/mol) can be deduced from the normalization conditions (25) and (26) as

(29) $$a_{j}=\sum_{k=q_{j}+1}^{n}n_{kj}M_{k}=-\sum_{k=1}^{q_{j}}\left(-n_{kj}\right)M_{k}\;.$$

In conformity with the usual sign convention the stoichiometric coefficients of reactants are counted as negative, to indicate that the corresponding substances are consumed during reaction. Now we are able to employ the rate Equation (22) to obtain a general expression for the reaction rate of each elementary reaction considered in our system

(30) $$J_{j}=k_{+1,j}\prod_{k=1}^{q_{j}}{\left[y_{k}\right]}^{n_{kj}}-k_{-1,j}\prod_{k=q_{j}+1}^{n}{\left[y_{k}\right]}^{n_{kj}}\;.$$

In contrast to the convention in Equation (29), there is no negative sign in the exponent of the product of the reactant results. In this way it is guaranteed that the exponents in our rate law are always positive as it was introduced in the general formalism (22). By means of relations (28)–(30) we are able to give a consistent representation of the generalized chemical reaction rates ${\bar{J}}_{j}$ and the scaled stoichiometric coefficients $v_{kj}$.

### 3.3. Formulation of the Problem

With the models (5) and (23) the temporal evolution of the concentration fields $c_{k}$ is captured by an interplay between the corresponding diffusive mass current $J_{k}$, which also contains the contributions of the elastic field, and a source term $\sum_{j=1}^{r}v_{kj}{\bar{J}}_{j}$ due to chemical reactions.

Thus, the evolution equations for chemically active multicomponent mixtures read

(31) $$\rho \frac{dc_{k}}{dt}=\nabla \cdot \left(\rho M\nabla \mu_{k}\right)+\sum_{j=1}^{r}v_{kj}{\bar{J}}_{j}\;\;\mathrm{in}\;\Omega \times \left[0,T\right]\;,$$

for k=1,…,n. The boundary conditions are given such that

(32) $$J_{k}\cdot n=0\;\;\mathrm{on}\;\partial \Omega \times \left[0,T\right]\;,$$

and

(33) $$\nabla c_{k}\cdot n=0\;\;\mathrm{on}\;\partial \Omega \times \left[0,T\right]\;,$$

where n is the unit outward normal on ∂Ω. As initial boundary condition, we employ

(34) $$c_{k}\left(x,0\right)=c_{k,0}\left(x\right)\;.$$

This reaction-diffusion problem definition comprises the competition of chemical reaction, elastic field and mass diffusion of a multicomponent system within generalized phenomenological evolution equations. A similar form of reaction-diffusion equations, expressing mass continuity but no deformation field, may also be found in [15] and in its original form in [6].

## 4. Computational Studies of Elastic Multicomponent Reaction-Diffusion Systems

In the following, we present two- and three-dimensional computational studies of binary and ternary systems in order to illustrate the effect of interfacial chemical reaction coupled with diffusion phenomena, such as phase separation, microstructural coarsening and swelling of the material. We will consider representative systems with non-dimensional parameters which suffice to illustrate the essential physics. It should be emphasized that simulations of multicomponent reaction-diffusion systems are not common. To our knowledge, there does not exist a computational simulation of real ternary or quaternary materials with chemically active phase-separation.

For our numerical solution we employ a NURBS based finite element approach for the spatial discretization and a classical Crank-Nicolson scheme for the temporal discretization. To get insight into the mathematical details of these discretization schemes the authors refer to their original papers on the treatment of similar Cahn-Hilliard type phase-field problems, cf. [16,17,18,19].

We would like to note that as a consequence of chemical reactions the solution scheme requires a fine resolution in time.

### 4.1. Evolution of a Ternary Reaction-Diffusion System

Although computational studies of binary reaction-diffusion systems can be performed, they are rather academic because chemical reactions usually involve multiphase mixtures, [20]. Therefore we simulate here a segregating ternary system subjected to interfacial chemical reactions and phase coarsening. Such a kind of chemical reaction was experimentally studied by Horiuchi et al. [21] to adjust morphologies in ternary immiscible polymer blends.

#### 4.1.1. Modelling the System

We consider an evolution scenario within an unstable ternary system consisting of species *A*, *B* and *C*, which is simultaneously subjected to the bimolecular reaction

(35) $$n_{A}A+n_{B}B\begin{array}{c} k_{+1}\\ ⇌\\ k_{-1} \end{array}n_{C}C\;,$$

where, according to our notation, $k_{+1}$ and $k_{-1}$ are the rate coefficients of the forward reaction $n_{A}A+n_{B}B\to n_{C}C$ and the backward reaction $n_{C}C\to n_{A}A+n_{B}B$. The corresponding reaction rate $J_{1}$ follows from Equation (22) as

(36) $$J_{1}=k_{+1}{\left[A\right]}^{n_{A}}{\left[B\right]}^{n_{B}}-k_{-1}{\left[C\right]}^{n_{C}}\;.$$

Following the ideas of [20] we consider a solid mixture which is quenched into a thermodynamically unstable state and simultaneously undergoes the elementary chemical reaction (35). This chemical reaction is regarded as an externally driven process induced, e.g., by irradiation.

The microstructural evolution of the considered ternary mixture is described by the temporal evolution of the mass fractions $c_{1}:=c_{A}$ and $c_{2}:=c_{B}$. The mass concentration $c_{3}:=c_{C}$ of component *C* follows from overall mass conservation as

$$c_{1}+c_{2}+c_{3}=1\;\Leftrightarrow \;c_{3}=1-\left(c_{1}+c_{2}\right)\;.$$

Then, the general evolution equations for multiphase reaction-diffusion systems (31) reduce to three ternary evolution equations

(37) $$\frac{\partial c_{1}}{\partial t}=\nabla \cdot \left(M\nabla \mu_{1}\right)-n_{A}M_{A}{\bar{k}}_{+1}c_{1}^{n_{A}}c_{2}^{n_{B}}+n_{A}M_{A}{\bar{k}}_{-1}{\left(1-\left(c_{1}+c_{2}\right)\right)}^{n_{C}},$$

(38) $$\frac{\partial c_{2}}{\partial t}=\nabla \cdot \left(M\nabla \mu_{2}\right)-n_{B}M_{B}{\bar{k}}_{+1}c_{1}^{n_{A}}c_{2}^{n_{B}}+n_{B}M_{B}{\bar{k}}_{-1}{\left(1-\left(c_{1}+c_{2}\right)\right)}^{n_{C}},$$

(39) $$\frac{\partial c_{3}}{\partial t}=\nabla \cdot \left(M\nabla \mu_{3}\right)+n_{C}M_{C}{\bar{k}}_{+1}c_{1}^{n_{B}}c_{2}^{n_{A}}-n_{C}M_{C}{\bar{k}}_{-1}c_{3}^{n_{C}}\;,$$

subjected to the mass conservation constraint. Since $c_{3}$ directly follows from the concentration fields $c_{1}$ and $c_{2}$, only Equations (37) and (38) need to be solved.

#### 4.1.2. Choice of Parameters

In our example we employ an averaged overall mobility M=M·I that captures the kinetics of the diffusion contribution within the evolution equations. The coefficients ${\bar{k}}_{+1}$ and ${\bar{k}}_{-1}$ denote scaled rate coefficients, which follow from the normalization conditions (25) and (26). The gradient coefficients of the individual phases $\kappa_{i}$ are considered to be approximately equal and therefore they are represented by an overall gradient coefficient κ. The same holds for the elastic moduli and so the elastic field has no effect in this first example. Summarizing, we employ the following dimensionless parameter set for the free-energy of the system:

$$M=1,\;\kappa =2.5\cdot 10^{-5},\;\theta \left(T\right)=0.35,\;\chi_{12}^{\left(0\right)}=\chi_{13}^{\left(0\right)}=\chi_{23}^{\left(0\right)}=1.2\;.$$

**Figure 2:**
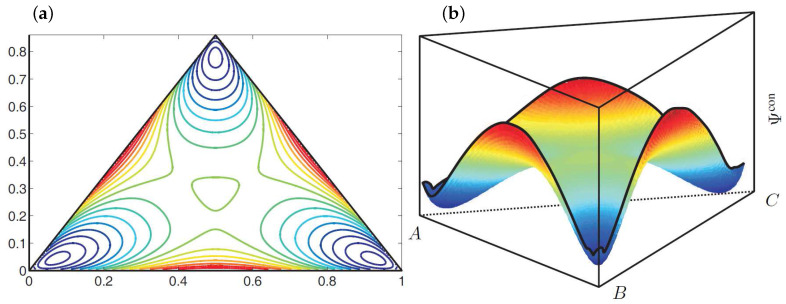
(**a**) Contour plot and (**b**) illustration of the shape of the configurational energy density $\Psi^{\mathrm{con}}$. The blue color indicates low energy values and the red color marks high energy values.

These material parameters represent a thermodynamically unstable mixture which will decompose into three equilibrium phases. The corresponding shape of the configurational energy within the Gibbs triangle is illustrated in Figure 2. Here the configurational energy has three local minima, which can be connected by a common tangent plane. The position of these minima characterizes the composition of the equilibrium phases.

For the parameters of the chemical reaction we choose, in the absence of experimental reference data, rather academic values. The stoichiometric coefficients are

(40) $$n_{A}=n_{B}=n_{C}=1\;.$$

Moreover, we assume that the molecular masses of substances *A* and *B* are similar $\left(M_{A}\approx M_{B}\right)$ as it is often the case for chemically reactive polymer blends. From a computational point of view the individual molar masses are simply included into the scaled rate coefficients

(41) $${\bar{k}}_{+1}^{\prime }:=M_{A}{\bar{k}}_{+1}=M_{B}{\bar{k}}_{+1}\;\;\mathrm{and}\;\;{\bar{k}}_{-1}^{\prime }:=M_{A}{\bar{k}}_{-1}=M_{B}{\bar{k}}_{-1}\;.$$

In our simulations we make use of equal forward and backward reaction rates and set ${\bar{k}}_{+1}^{\prime }={\bar{k}}_{-1}^{\prime }={\bar{k}}^{\prime }$; we will vary this parameter later. We would like to mention that our model is capable to also reproduce other settings for chemical reactions.

#### 4.1.3. Results of the Two-Dimensional Simulations

**Figure 3:**
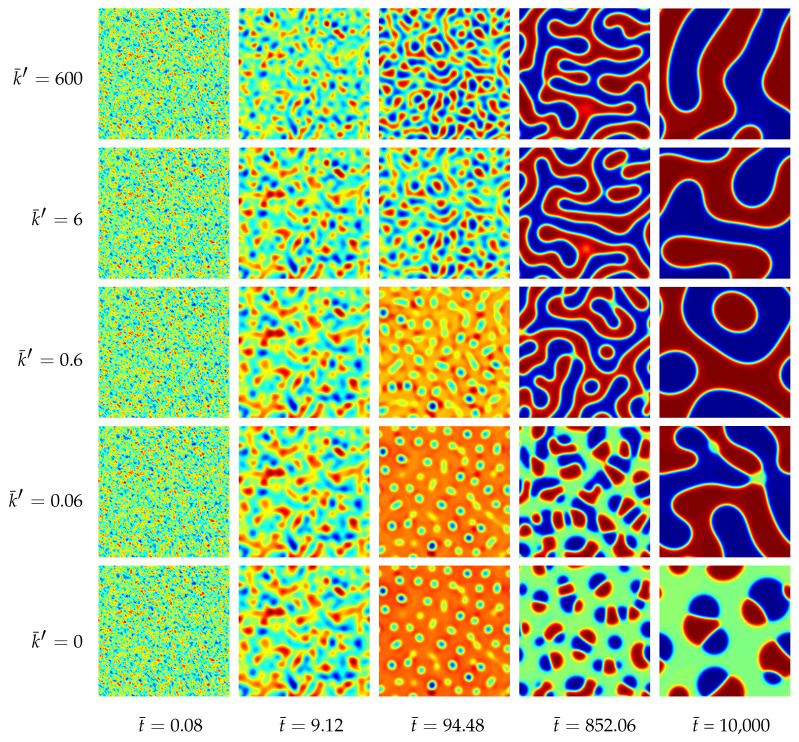
Morphology evolution in a ternary reaction-diffusion system at early stages for different reaction rates ${\bar{k}}^{\prime }$ and at different stages of evolution $\bar{t}$. The instances of the morphological snapshots correspond to the times marked with circles in Figure 4. Color coding: $c_{A}$ (red), $c_{B}$ (blue), $c_{C}$ (green).

**Figure 4:**
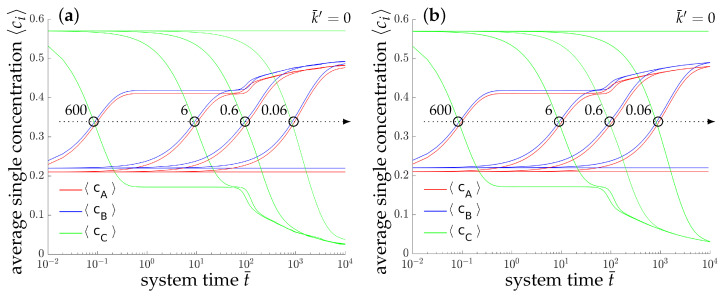
Temporal evolution of the averaged concentrations $\langle c_{A}\rangle$ (blue), $\langle c_{B}\rangle$ (red) and $\langle c_{C}\rangle$ (green) of the chemical species *A*, *B* and *C*, respectively, for the two-dimensional simulation (**a**) and the three-dimensional simulation (**b**). The maximum deviation between the average 2D and 3D concentrations is less than $6.2\times 10^{-3}$ for $\bar{t}<10^{3}$.

The morphological evolution of our representative ternary reaction-diffusion system is displayed in Figure 3 for different values of ${\bar{k}}^{\prime }$. All simulations start from a homogeneous state with slightly perturbed concentration profiles $c_{A,0}=c_{1,0}\approx 0.22$, $c_{B,0}=c_{2,0}\approx 0.21$ and $c_{C,0}=c_{3,0}\approx 0.57$, i.e., the system is arranged outside the chemical equilibrium. As known from other diffusion problems, the system’s microstructural evolution is dominated by the tendency to reach a stable state in the course of time. This trend is illustrated in the overall concentration evolution of the substances *A*, *B* and *C* in Figure 4. We observe here a complete rearrangement of the system’s composition. While the overall concentration of the reaction product *C* decreases, the concentrations of *A* and *B* increase in time. The equilibration of chemical reactions takes place in a time interval which strongly depends on the reaction rates, e.g., between $\bar{t}=0$ up to t=0.5 for the fastest reaction with ${\bar{k}}_{+1}^{\prime }={\bar{k}}_{-1}^{\prime }=600$, smaller reaction rates need longer times to reach a chemical equilibrium.

#### 4.1.4. Results of the Three-Dimensional Simulations

**Figure 5:**
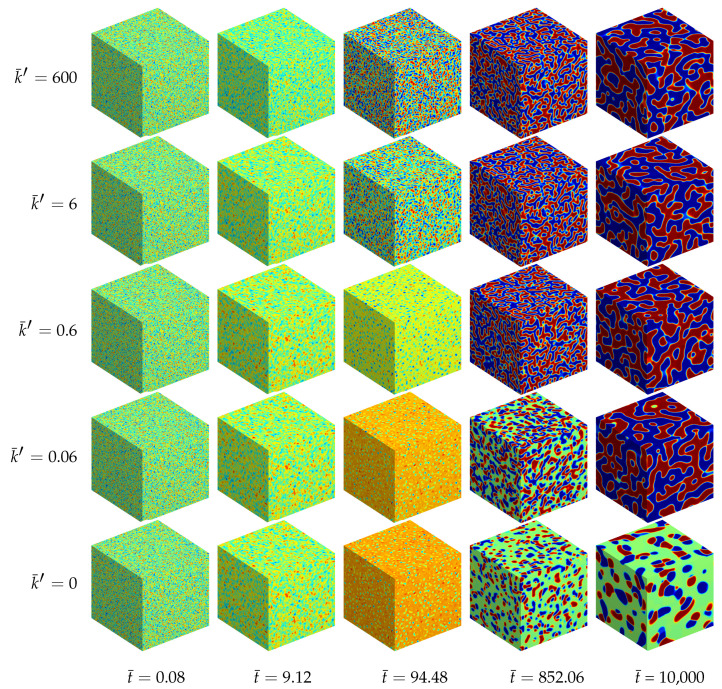
Morphology evolution in a ternary reaction-diffusion system at early stages for different reaction rates ${\bar{k}}^{\prime }$ and at different stages of evolution $\bar{t}$. The instances of the morphological snapshots correspond to the times marked with circles in Figure 4. Color coding: $c_{A}$ (red), $c_{B}$ (blue), $c_{C}$ (green).

Additionally, a ternary reaction-diffusion system with the same material parameters as above has been simulated in a fully three-dimensional setting. The morphology evolution is displayed in Figure 5. The computed unit cube has periodic boundary conditions with no external flux, i.e., the initial state with $c_{A}\approx 0.22$, $c_{B}\approx 0.21$ and $c_{C}\approx 0.57$ is quenched in a thermodynamically unstable state. Therefore, the initially homogeneous mixture rearranges for all analyzed systems into chemical equilibrium at first. Next, separation into three equilibrium phases takes place where the final morphology, as well as the time till a stable decomposed state is reached, depend on the reaction rate, cf. Figure 4.

For the chemically active mixtures the evolving microstructure is dominated by a lamellar pattern. This structure follows from two equivalent *A*-rich and *B*-rich major phases. In the computational micrographs shown in Figure 5 the *A*-rich phase is displayed in blue and the *B*-rich phases are red. The third *C*-rich equilibrium phase (green domains) represents here the minor phase, which accumulates at the interfacial regions between the *A*- and *B*-rich lamellae. The reason for this accumulation of *C*-type phase at the interfaces, which leads to an encapsulation of the major phases, is the chemical reaction. Since the bimolecular reaction (35) requires matter of *A* and *B* in order to produce *C*, the chemical reaction is concentrated at the interfacial regions where *A* and *B* occur simultaneously. In the bulk regions, where there is a persistently high amount of *A* and a lack of *B* and vice versa, the chemical reaction is complicated by the missing other reactant. This special kind of morphology formation was experimentally observed by [21].

If chemical reactions are absent, the resulting morphology is completely different. In this case we observe two cobblestone-like phases embedded in a continuous matrix phase, see also [22].

#### 4.1.5. Parametric Studies

**Figure 6:**
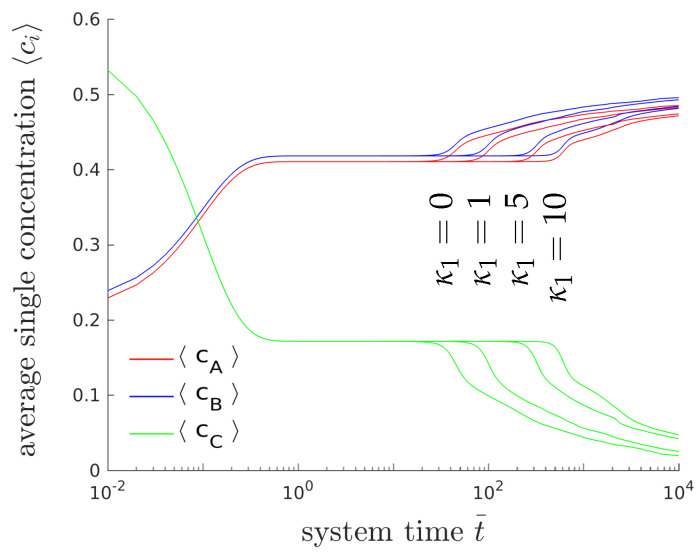
Temporal evolution of the averaged concentrations $\langle c_{A}\rangle$ (blue), $\langle c_{B}\rangle$ (red) and $\langle c_{C}\rangle$ (green) of the chemical species *A*, *B* and *C*, for different values of gradient energy coefficient $\kappa_{1}$ using ${\bar{k}}^{\prime }=600$.

Figure 6 shows the temporal evolution of the overall concentrations $c_{A}$, $c_{B}$ and $c_{C}$ for different interfacial energy contributions. Only the gradient energy coefficient of the first phase ($\kappa_{1}$) is varied. Please note that the gradient energy coefficient κ is related to the surface energy density γ and the thickness *l* of the interfacial layers between the domains by κ=γl. The higher the surface tension is, the longer it will take until the system branches from the plateau and reaches chemical equilibrium. The formation of lamellar microstructures, however, is not affected by the surface tension. Please note that due to the modification of only one of three gradient energy coefficient the whole dynamical behavior changes and thus, it is hard to compare the obtained results with classical binary decomposition problems, where a high surface tension accelerates the process of coarsening.

As a further exemplary study of pattern formation, an externally driven chemical reaction is sketched. Here we prescribe a locally varying reaction rate which may be induced, e.g., by a proper catalyzer. To this end, we define domain dependent rate parameters ${\bar{k}}_{+1}^{\prime }={\bar{k}}_{-1}^{\prime }={\bar{k}}^{\prime }$ with

$${\bar{k}}^{\prime }\left(x,y\right)=\exp\left(-50\left|\left(x-½)(y-½\right)\right|\right)\cdot 600\;.$$

**Figure 7:**
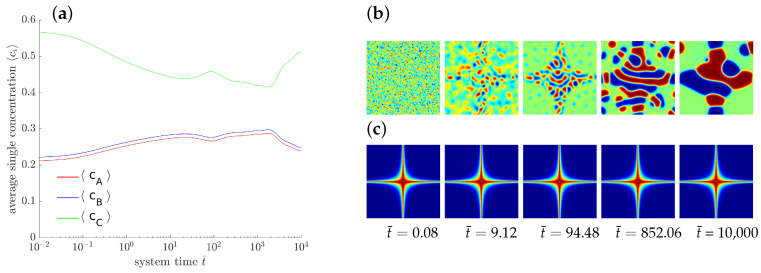
Evolution of a ternary reaction-diffusion system for locally varying ${\bar{k}}^{\prime }$. The overall concentrations (**a**) and the corresponding morphology are presented at different stages (**b**) with the corresponding spatial distribution of ${\bar{k}}^{\prime }$ (**c**).

The corresponding distribution of ${\bar{k}}^{\prime }$ is displayed in Figure 7c with red and blue indicating high and low values, respectively. The temporal evolution of the averaged concentrations indicates, that neither a chemical equilibrium nor a stable state is reached. The evolving microstructure is controlled by the rate of the chemical reactions.

Further computational studies with different initial settings have shown that the typical laminar structures, where one phase is encapsulated by a thin layer of another phase, are invariant under modifications of the initial concentration. This is not the case in classical, non-reactive phase-separating mixtures where the microstructural formation commonly depends on their initial configuration. As Anders and Weinberg have shown in [19] in a classical binary system with initial concentration $c_{A}\approx c_{B}$ a lamellar structure forms. In contrast for significantly different initial concentrations $c_{A}$ and $c_{B}$ spherical islands within a matrix appear. These results support the idea that chemical reactions can be employed to force the morphology of multicomponent systems out of its original arrangement into a capsule formation or other types of desired microstructural formations.

### 4.2. Evolution of Elastic Phase-Separating Systems

In the following we present the evolution of multi-component systems with particular emphasis on the effect of the elastic field. The two investigated specimens have a cylindrical shape with radius *r* and length *l*.

#### 4.2.1. A Rod under to Incoming Flux

A slim rod, consisting of a uniform elastic material *A*, is subjected to an incoming diffusive flux from one side. For symmetry reasons only one quarter of the cylindrical rod with 2 µm in diameter and 10 µm edge length is meshed, using 32×256 hexahedral NURBS elements of polynomial order p=2. A normal mass flux $j_{n}$ of component *B* is prescribed at the top end, while the lateral surface and the bottom are flux-free, $j_{n}=0$. The mechanical boundary conditions are chosen in such a way, that only rigid-body movements are prevented, otherwise the rod is free to deform. The normal flux $j_{n}=64\times 10^{-3}$ is applied for a system time period of t=2800, and after its removal the system will evolve until t=5000.

The initial concentration is $c_{A}=0.75$, $c_{B}=0.25$ and $c_{C}=0$. The system follows the evolution Equations (31) with configurational energy (11) and overall equal material parameters,

$$M=c_{A}c_{B},\;\kappa =10^{-10},\;\theta \left(T\right)=1,\;\chi_{12}^{\left(0\right)}=\chi_{13}^{\left(0\right)}=\chi_{23}^{\left(0\right)}=2.5\;.$$

As we have shown in our previous examples, a bimolecular chemical reaction (35) can only take place at the interface between phases *A* and *B*. Therefore the chemical phase *C* has basically no effect on the microstructural evolution here and we can proceed setting $c_{C}\approx 0$. It follows $c_{B}\approx 1-c_{A}$; however, the mass of the system is not conserved. The equilibrium phases are found at $c_{B}=0.145$ and $c_{B}=0.855$.

The elastic energy (10) employs concentration dependent material parameters. Thus the concentration independent elastic moduli are set within (17) and (18) to be $K_{A}=G_{A}=10$ GPa and $K_{B}=G_{B}=9$ GPa, respectively. Please note that in this model the material softens with rising concentration $c_{B}$; an opposite model is possible as well. Furthermore, we adapt the International Union of Pure and Applied Chemistry (IUPAC) definition of standard conditions for temperature and pressure, i.e., T=298.15 K, a universal gas constant R=8.314 J/(mol K) and employ a maximum concentration $y_{0}=2.29\times 10^{4}$ mol/m^3^ [23] (p. 339).

**Figure 8:**
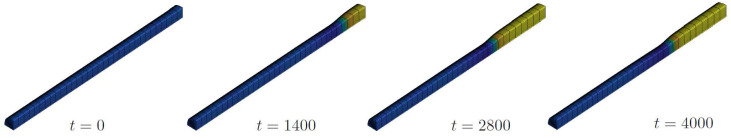
Diffusion of component *B* in a rod of material *A* at different times.

Figure 8 shows the distribution of the concentration $c_{B}$ at different system times. The amount of incoming flux is rather low, so that the concentration evolves directly into segregated phases of minimum free energy. The interface between the phases moves towards the free end. Once the flux stops, the system will evolve to the equilibrium state which, in our case, does not bring significant changes.

The two phases can easily be recognized by its deformation. The top side is loaded and swollen whereas the remaining rod is close to the initial concentration. The rod is free to expand and, therefore, stresses are observed only in the transition region between the two phases. The major effect of the stresses concerns the equilibrium concentrations. Here we observe a stable state at $c_{A}=0.145$, $c_{B}=0.855$ and $c_{C}=0$.

#### 4.2.2. Phase Decomposition in an Elastic Rod

In a final example we simulate a rod with r=1 µm and l=10 µm each. The full rod is now meshed with 25×25 finite elements. Note that there is a homeomorphism between the topological spaces of the rod and a cube and by a careful geometrical modelling of the NURBS based open-knot system we made sure, that there are no spurious concentration or stress effects at the edges of the descendant cube. In particular, the initial mesh configuration has been verified to be stress free.

For the configurational energy the material parameters are chosen as in the previous example. Now there is no external flux applied. The rod is supported along its axis; in radial direction it is free to deform and the flux-free boundary condition (32) holds everywhere.

**Figure 9:**
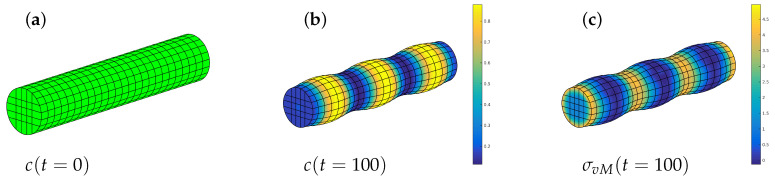
Phase decomposition in an elastic rod: initial state (**a**) concentration distribution after 100 time steps (**b**), and corresponding von Mises stress (**c**).

We choose an initial concentration of $c_{A}\approx c_{B}=0.5$ and let the system evolve in time. The initial state has a uniform concentration and a certain swelling which, of course, is homogeneous. The chemical reaction rate is ${\bar{k}}^{\prime }=0$ and so we set $c\equiv c_{B}=1-c_{A}$. The result can be seen in Figure 9. Then the concentration changes toward the equilibrium phases and the phases decompose. As above, the stress-free equilibrium phases are $c_{\alpha }=0.145$ and $c_{\beta }=0.855$ but the latter reduces as a consequence of the elastic field. The swelling changes accordingly and we get differently deformed regions of the rod, indicating high and low values of concentration. The elastic stresses are also displayed and we see that the maximum von Mises stress of about $5RTy_{0}$ (yellow in Figure 9, left) is located at places with minimum concentration $c_{B}$ whereas at high values of $c_{B}$ the material is relaxed. Although a quantitative analysis would require reliable material parameters, this example illustrates nicely the interplay of elasticity and phase decomposition.

## 5. Summary

The functional properties of multi-component materials are often connected with chemical reactions. To enable computational simulations and structural optimization of such materials, we presented a material model for reactive multi-component solid mechanical systems. The physical processes which are assumed to contribute are: mechanical deformation of the system, phase decomposition and coarsening, chemical reactions between the multiple components and the energetic forces associated with the elastic field of the solid. After deriving the chemo-mechanical model in a thermodynamically consistent way, for the first time numerical simulation of reactive, ternary phase-separating systems undergoing large elastic deformations are presented. The computations are performed with a NURBS based finite element analysis.
